# The Prognostic Value of Sarcopenia in Clinical Outcomes in Cervical Cancer: A Systematic Review and Meta‐Analysis

**DOI:** 10.1002/jcsm.13674

**Published:** 2025-01-11

**Authors:** Fang Wang, Hongnan Zhen, Kang Yu, Pengju Liu

**Affiliations:** ^1^ Department of Clinical Nutrition Peking Union Medical College Hospital Beijing China; ^2^ Department of Radiology Peking Union Medical College Hospital Beijing China

**Keywords:** meta‐analysis, muscle loss, progression free, sarcopenia, survival, survival cervical cancer

## Abstract

**Background:**

Sarcopenia is a condition characterized by inadequate muscle and function decline and is often associated with ageing and cancer. It is established that sarcopenia and muscle loss occurred during treatment are associated with the clinical outcomes of patients with cancer. This systematic review and meta‐analysis aims to evaluate the association between sarcopenia at pretreatment and during treatment and overall survival or disease progression in patients with cervical cancer.

**Methods:**

The Web of Science, Embase, Medline and Cochrane Library databases were searched until 4 July 2024. Studies evaluating the prognostic effect of muscle mass at pretreatment or muscle change during treatment on survival or disease progression for patients with cervical cancer were included. Study quality was evaluated with the Newcastle–Ottawa Scale (NOS). Forest plots and summary effect models were used to show the effect size of sarcopenia on clinical outcomes.

**Results:**

The search strategy yielded 1721 studies in four databases. Eleven and seven studies were included in the quantitative analysis of pretreatment sarcopenia and muscle change on clinical outcomes, respectively. A total of 1907 patients underwent pretreatment muscle assessment, but 1016 were monitored for muscle changes; however, none of the studies involved measures of muscle strength or function. Meta‐analysis showed a significant association between pretreatment sarcopenia and OS [hazard ratio (HR) 1.58, 95% confidence interval (CI): 1.16–2.14, *p* = 0.003] and PFS (HR 1.63, 95%CI 1.16–2.29, *p* = 0.005) according to data of univariate analysis. In the meta‐analysis of the multivariate data, pretreatment sarcopenia remained associated with poor OS (HR 3.09, 95% CI: 2.07–4.61, *p* < 0.00001) and PFS (HR: 1.55, 95%CI 1.06–2.28, *p* = 0.03). Additionally, muscle loss was significantly associated with OS (HR 5.18, 95%CI 3.54–7.56, *p* < 0.00001) and PFS (HR 2.62, 95%CI 1.63–4.22, *p* < 0.00001). Subgroup analysis showed that the association between pretreatment sarcopenia and OS, as well as PFS, was influenced by muscle mass measurements and cut‐off values, whereas muscle loss consistently predicted worse OS and PFS when stratified by varying degrees of reduction. The NOS scores of all included studies were ≥ 6.

**Conclusions:**

Pretreatment sarcopenia and muscle change during treatment are significantly associated with both overall survival and disease progression. Therefore, muscle assessment and monitoring should be conducted for appropriate diagnosis and intervention to improve clinical outcomes in patients with cervical cancer.

## 1 Background

Cervical cancer (CC) ranks as the fourth most common cancer and the fourth leading cause of cancer‐related deaths among women, with 604 000 new cases and 342 000 deaths globally in 2020 [1]. Despite advancements in prevention and early screening, CC remains prevalent, particularly in less developed countries, imposing a significant burden on public health systems worldwide. Identifying specific predictors of survival outcomes can enhance decision‐making and risk stratification, leading to improved clinical outcomes for patients with CC. Moreover, in personalized medicine, efforts are also focusing on exploring new therapeutic targets and assessing patient's physical ability to undergo treatment.

Skeletal muscle, the largest organ in the human body, plays a crucial role in energy and nutrient metabolism. Inadequate muscle mass, known as sarcopenia, is commonly associated with ageing but also has a high incidence in patients with cancer, regardless of age [2]. It is an important feature of cancer‐related malnutrition and cachexia [3]. Recent studies in oncology have emphasized the significant impact of muscle mass on clinical outcomes, including perioperative complications, disease progression and overall survival [4, 5]. Although CC does not directly affect the gastrointestinal tract, anticancer treatment can induce gastrointestinal reactions and lead to a reduction in dietary intake. Combined with the abnormal metabolism of tumours, this predisposes patients to develop sarcopenia, which has an incidence rate of 33.3%–51.8% [6]. Methods for muscle mass assessment include dual‐energy x‐ray absorptiometry, bioelectrical impedance analysis, magnetic resonance imaging and computed tomography (CT), with CT considered the gold standard [7]. As CT scans are routinely used for diagnostic and evaluation purposes for patients with cancer, they are the most frequently used method for quantifying skeletal muscle mass in this population. However, the diagnosis criteria and cut‐off values for sarcopenia remain undefined.

Given the limited sample sizes of individual clinical studies, a meta‐analysis is essential to further investigate the influence of sarcopenia on clinical outcomes in patients with CC. Whereas some previous meta‐analyses have explored the relationship between muscle mass and clinical outcomes in gynaecologic malignancies [8, 9, 10, 11], specific data for CC have been lacking. These studies only included three to seven articles about CC published before 2022. With several new trials published between 2022 and 2024, an update to these findings is necessary. Furthermore, despite consistent principles of anticancer treatment and radiotherapy regimens, the degree of muscle loss experienced during treatment varies and may also impact clinical outcomes.

Therefore, with more up‐to‐date research, this study aims to quantitatively summarize the current evidence and achieve a better understanding of the clinical significance of sarcopenia and muscle changes during treatment concerning survival and disease progression in patients with CC, which will help improve risk prediction models and develop targeted nutrition interventions in the future.

## 2 Materials and Methods

### 2.1 Search Design

This systematic review and meta‐analysis were conducted based on the Preferred Reporting Items for Systematic Reviews and Meta‐Analyses (PRISMA) [12]. This study follows the format of an evidence‐based medical literature search to summarize the effect of muscle mass on progression‐free survival (PFS), disease‐free survival (DFS), distant failure‐free survival (DFRS) and overall survival (OS) in patients with CC. Databases searched included Web of Science, Embase, Medline and Cochrane Library, with the language restriction being English. The search was conducted from inception up until 4 July 2024. The cervical cancer‐related search terms, sarcopenia‐related search terms and muscle‐related terms were used. Further details regarding the search strategy are provided in Table S1. Reference lists of included studies were manually examined to obtain additional relevant data.

### 2.2 Study Selection

The following types of studies were included (1) studies of patients pathologically diagnosed with CC; (2) studies reporting pretreatment and/or changes in muscle; (3) prospective or retrospective studies; and (4) studies reporting hazard ratios (HRs) and 95% confidence intervals (95%CI) of OS, PFS, DFS or (DFRS) of patients with CC. Studies were excluded if they were (1) duplicate research; (2) studies involving patients with non‐CC or combined with other types of cancer; (3) animal or cell experiments; (4) studies with incomplete data; or (5) case reports, reviews, conference abstracts, expert opinions and guidelines. Two researchers (F.W. and P.J.L.) searched the databases independently. If disagreements or uncertainty occurred, the results were discussed with a third researcher to evaluate and reach an agreement.

### 2.3 Data Extraction

Data extraction was also conducted by F.W. and P.J.L. independently. Extracted data included first author's name, year of publication, journal, sample size, type of pathology, cancer stage, muscle measurement methods, cut‐off values for sarcopenia diagnosis or low muscle mass, prevalence of sarcopenia and correlated HRs of OS with 95% CI. HRs were extracted from univariate and multivariate analysis, otherwise from software to extract from the survival analysis charts [13].

### 2.4 Quality Assessment

Quality assessment was conducted according to the Newcastle–Ottawa Scale (NOS), and studies with scores of ≥ 6 were regarded as high quality [14]. F.W. and P.J.L. evaluated the NOS independently; in case of disagreements, the results were reviewed by a third researcher to reach an agreement.

### 2.5 Statistical Analysis

In this meta‐analysis, forest plots were used to show the effect size of the studies and visualize the results. HR > 1 was generally considered a risk factor, and *p* < 0.05 was considered statistically significant. *I* ^2^ and χ^2^ statistics were used to test the heterogeneity among studies. If *I* ^2^ ≥ 50% or χ^2^ < 0.10, heterogeneity was considered significant; if *I* ^2^ < 50%, heterogeneity was acceptable.

The log HR with standard errors (SE) was considered the effect size to pool in summary, with the pooled results value showing the influence of sarcopenia or muscle loss during treatment on CC prognosis. Sensitivity analysis of the included studies was conducted by removing one study at a time to test the reliability. Funnel plots and Egger and Begg's tests were applied for publication bias assessment; no publication bias was considered if *p* > 0.05. This meta‐analysis was performed in Review Manager (RevMan V5.4; Copenhagen, Denmark), and Egger and Begg's tests were conducted by the STATA 12.0 software package.

## 3 Results

### 3.1 Search Results

**FIGURE 1:**
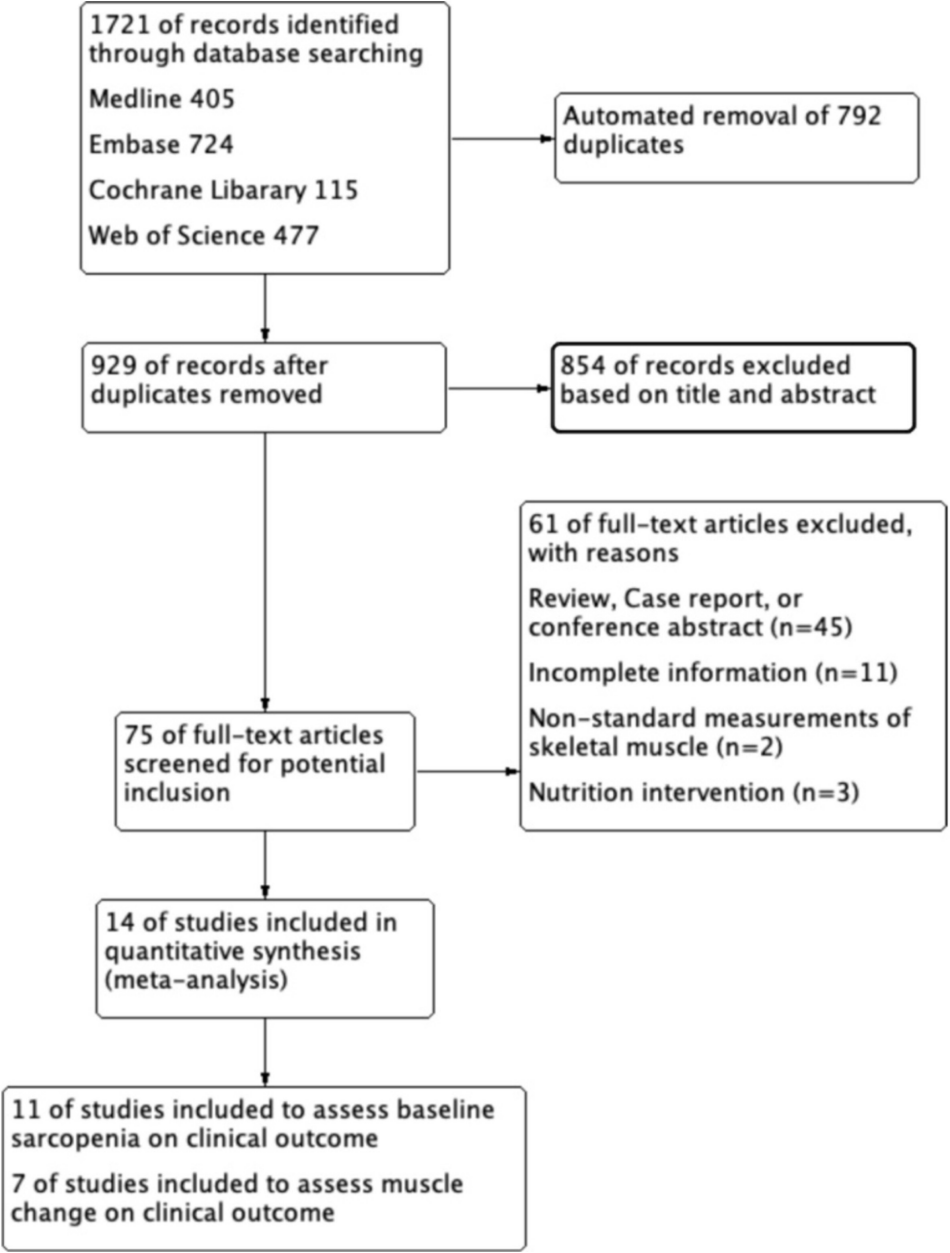
The flow diagram indicates the process of study selection.

The initial search through the electronic databases yielded 1721 studies. After removing duplicates (*n* = 792), 929 studies were scanned with titles and abstracts. Of these, 854 were further excluded being irrelevant, leaving 75 studies for full‐text review. Subsequently, 61 studies were excluded for not meeting the inclusion criteria. Finally, 14 studies were included in this meta‐analysis (Figure 1).

### 3.2 Included Studies and Characteristics of Patients

Table 1 shows the main characteristics and details extracted from the included studies. The total number of patients was 2020. Of these, 12 studies [15, 16, 17, 18, 19, 20, 21, 22, 23, 24, 25, 26] (*n* = 1907 patients) examined the relationship between pretreatment muscle mass and clinical outcomes, and 7 studies [15, 18, 21, 22, 24, 27, 28] (*n* = 1016 patients) investigated the relationship between muscle mass changes during treatment and clinical outcomes. None of the studies performed muscle strength or function assessment. The mean age ranged from 50.5 to 63.0 years, and the sample size ranged from 40 to 278. Most patients were from Asian countries, with a few from Mexico and Germany. All studies were retrospective except for one prospective study by Sánchez et al. [27].

**TABLE 1:** Study characteristics and basic information of included studies.

Author, year	Country	*n*	Age	FIGO	Treatment	Muscle mass measurement	Cut‐off value	Muscle function/strength	Prevalence of sarcopenia	Outcome	NOS
Lee, 2022 [15]	Taiwan, China	133	Median 53 (46–61)	IB‐IIA	Surgery + RT/CCRT	SMI	Lowest tertile, 38.5 cm^2^/m^2^	Absent	NA	DFS, OS	8
Yoshikawa, 2020 [16]	Japan	40	56.9 ± 13.4	IV	CCRT	PMI	ROC, 3.72 cm^2^/m^2^	Absent	58.0%	OS	6
Han, 2021 [17]	Korea	306	51.5 ± 11.3	IB1‐IIA2	Surgery + RT/CCRT	SMI, volumetric muscle	Fearon, first quartile, 181.5 cm^3^/m^3^	Absent	46.1%	PFS, OS	8
Kiyotoki, 2018 [18]	Japan	60	52.8 ± 14.3	I‐IV	CCRT	SMA and IM	Mean, 90.29 cm^2^, 10.07 cm^2^	Absent	55.0%	OS, PFS	6
Matsuoka, 2019 [19]	Japan	236	58.8 ± 18.2	IB‐IVA	CCRT	SMI	ROC, 36.55 cm^2^/m^2^	Absent	NA	PFS, OS	6
Aichi, 2023 [20]	Japan	92	56.0 ± 13.4	III	CCRT	SMI	ROC, 35.6 cm^2^/m^2^	Absent	26.1	PFS, OS	7
Lee, 2020 [21]	Taiwan, China	278	62.5 ± 5.8	IB‐IVA	CCRT	SMI	Lowest tertile, 36.3 cm^2^/m^2^	Absent	33.0%	DRFS	8
Lee, 2018 [22]	Taiwan, China	245	63.0 ± 12.7	IB‐IVA	CCRT	SMI	Martin, 41 cm^2^/m^2^	Absent	51.8%	OS	8
Nattenmüller, 2018 [23]	German	104	62.9 ± 13.5	I‐IV	RT/CT/CCRT	SMI	Martin 41 cm^2^/m^2^	Absent	34.2%	OS	6
Abe, 2022 [24]	Japan	83	Median 60 (33–80)	I‐III	CCRT	SMI	Martin, 38.5 cm^2^/m^2^	Absent	30.1%	PFS, OS	7
Guo, 2023 [25]	Mainland, China	196	Mean 52	IIB‐IIIC	CCRT	SMI	Martin, 41 cm^2^/m^2^	Absent	45.4	OS	6
Kise, 2022 [26]	Japan	134	Median 49 (22–72)	IB‐IVA	CCRT	PMI	Hamaguchi, 3.92 cm2/m2	Absent	50.7%^a^	PFS, OS	6
Sánchez, 2019 [27]	Mexico	55	50.5 ± 11.4	II‐IV	CCRT	SMI	Prado, 38.5 cm^2^/m^2^	Absent	33.3%	PFS, OS	7
Fu, 2024 [28]	Mainland, China	58	56.0 ± 9.7	I‐IV	CCRT	PMA	NA	Absent	NA	OS, PFS	5

Abbreviations: CCRT, concurrent chemoradiotherapy; DFS, disease‐free survival; DRFS, distant recurrence‐free survival; FIGO, International Federation for Gynecologic Oncology; IM, iliopsoas muscle area; NOS, Newcastle–Ottawa Scale; OS, overall survival; PFS, progression‐free survival; PMA, paravertebral muscle area; PMI, psoas muscle index; RT, radiotherapy; SMA, skeletal muscle area; SMI, skeletal muscle index.

^a^ The prevalence of sarcopenia was calculated according to data from the study (68 patients with PMI < 3.92 cm2/m2).

FIGO (International Federation for Gynecologic Oncology) stage across all studies ranged from I to IV, whereas study by Yoshikawa et al. [16] only included patients of stage IV and studies by Lee et al. [15] and Han et al. [17] only included patients of early stage. All patients underwent concurrent chemoradiotherapy (CCRT), radiotherapy only or surgery. Follow‐up times varied from 14 to 62.7 months. Most included studies applied SMI for muscle mass assessment, but Kiyotoki et al. [18] used skeletal muscle area (SMA) and iliopsoas muscle area, Yoshikawa et al. [16] used psoas muscle index (PMI), Kise et al. [26] used PMI and SMI, and Han et al. [17] used both SMI and volumetric SMI as muscle assessment methods. For sarcopenia diagnosis, Yoshikawa et al. [16], Matsuoka et al. [19], and Aichi et al. [20] adopted ROC curves to determine the cut‐off value, Lee's two studies [18, 24] adopted the lowest tertile, Kise et al. [26] and Sánchez et al. [27] adopted the Prado criteria [29], Han et al. [17] applied the definition for cancer cachexia by Fearon et al. [3], and the remaining studies used the Martin criteria [30]. Ten studies showed the prevalence of pretreatment sarcopenia in patients with CC ranging from 26.1% to 63.9%. The lowest prevalence was reported by Aichi et al. [20], with a cut‐off value of 35.6 cm^2^/m^2^, which was lower than the commonly used ones of Fearon et al. [3], Prado et al. [29] and Martin et al. [30], and the highest prevalence was the study that used SMD as a measurement [22].

### 3.3 Pretreatment Muscle Mass and Clinical Outcomes

#### 3.3.1 Association of Pretreatment Sarcopenia With Overall Survival

**FIGURE 2:**
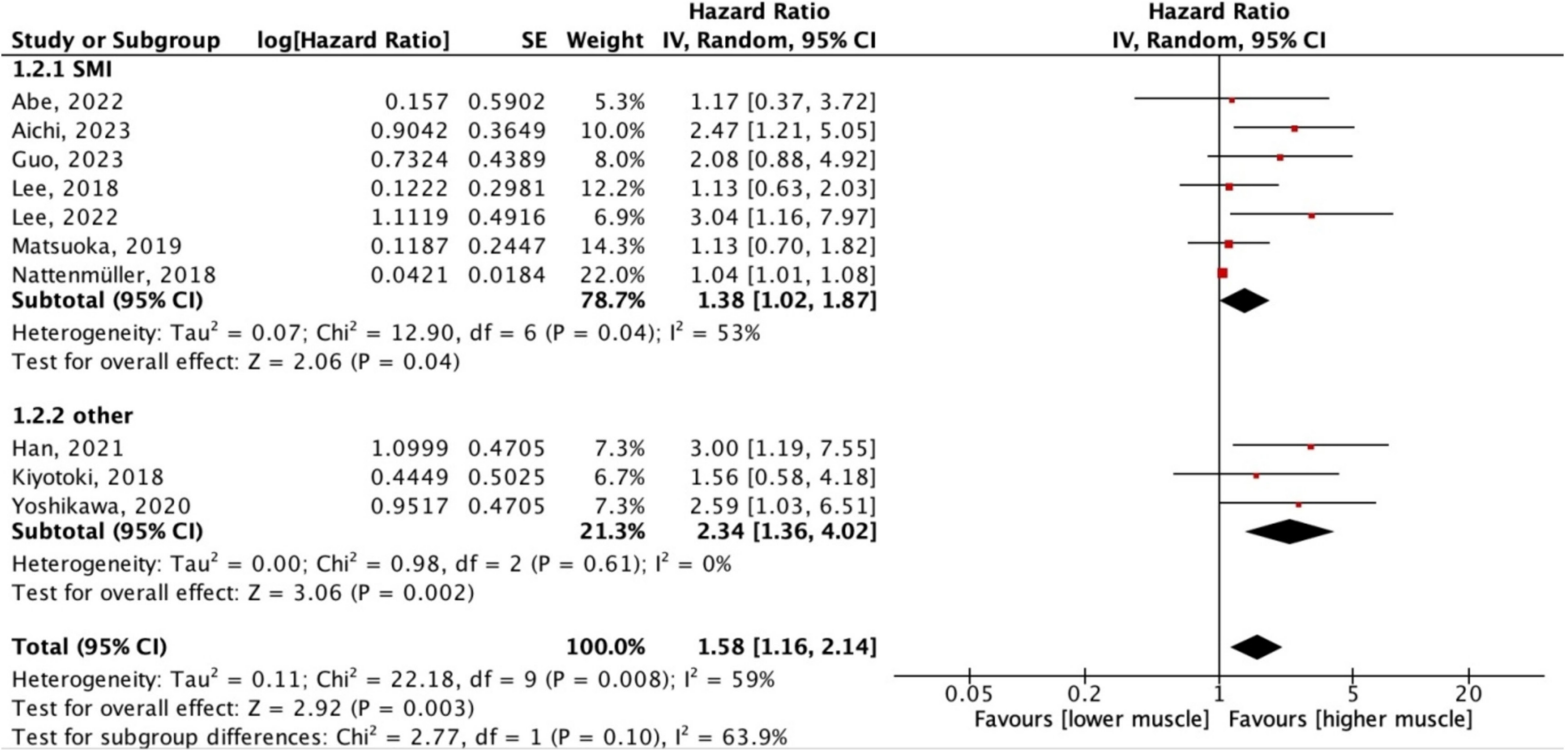
Meta‐analysis of univariate data: the effect of pretreatment sarcopenia on overall survival. CI, confidence interval; df, degree of freedom; IV, inverse variance; SE, standard error.

There are 10 studies reporting univariate regression analysis data on the influence of pretreatment sarcopenia on OS [15, 16, 17, 18, 19, 20, 22, 23, 24, 25], among them being Yoshikawa et al. [16], Han et al. [17] and Kiyotoki et al. [18], adopting PMI, SMA and volumetric SMI for muscle mass assessment, and the remaining adopting SMI. The meta‐analysis indicated the overall effect of sarcopenia on OS was significant (HR 1.58, 95% CI: 1.16–2.14, *p* = 0.003) (Figure 2). Statistical consistency between the compared HRs and 95% CIs was evaluated with the χ^2^ and *I* ^*2*^ tests, which returned a χ^2^ 0.04 and an *I* ^*2*^ of 59%, indicating the heterogeneity between studies, and the random effects model was chosen. The subgroup analysis according to muscle mass measurement showed that consistent results regardless of the muscle mass measurement methods used (volumetric muscle, SMA and PMI). When SMI was used for muscle mass assessment, the overall effect of sarcopenia on OS remained significant (HR 1.38, 95% CI: 1.02–1.87, *p* = 0.04). Heterogeneity within this subgroup remained large, potentially due to varying cut‐off values. The subgroup analysis according to the SMI cut‐off value showed that if SMI ≤ 38.5 cm^2^/m^2^ was used as the cut‐off value for sarcopenia diagnosis, the pretreatment sarcopenia was not a significant indicator for poor OS (HR 1.31, 95% CI: 0.92–1.88, *p* = 0.14). If SMI ≤ 41 cm^2^/m^2^ was used, the combined result was 1.10, 95% CI: 0.88–1.38, and *p* value was 0.4.

Matsuoka et al. [19] and Abe et al. [24] used both SMI and PMI as indicators of muscle assessment; thus, for studies using PMI, we combined the data and found that low PMI was not a predictor of OS (HR 1.66, 95% CI 0.79–3.51, I^2^ = 55%, *p* = 0.18) (Figure S1).

**FIGURE 3:**
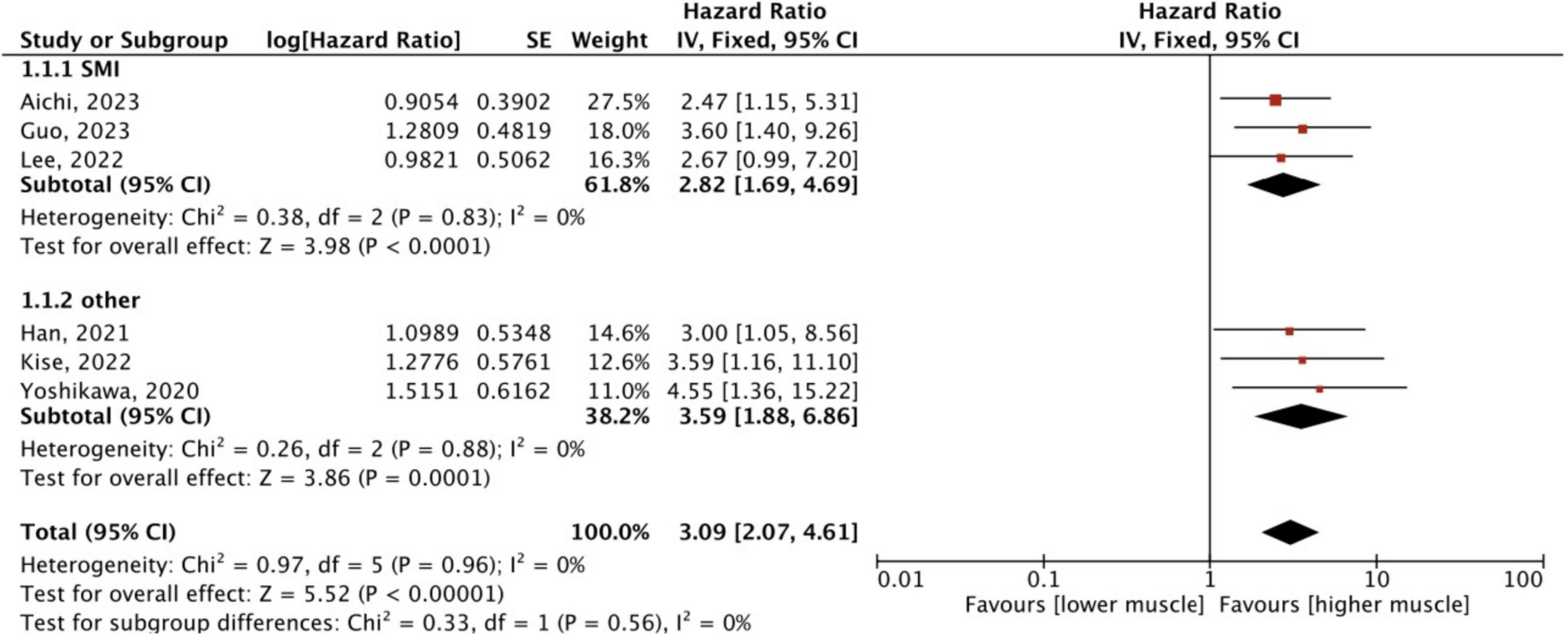
Meta‐analysis of multivariate data: the effect of pretreatment sarcopenia on survival. CI, confidence interval; df, degree of freedom; IV, inverse variance; SE, standard error.

In the meta‐analysis of the multivariate data retrieved from six studies [15, 16, 17, 20, 25, 26], there was also a significant association between sarcopenia and OS (HR 3.09, 95% CI: 2.07–4.61, *p* < 0.00001) no matter the muscle mass measurements used (Figure 3). A χ^2^‐test *p* value of 0.97 and an *I* ^*2*^ of 0% indicated consistency between studies for this association.

#### 3.3.2 Association of Pretreatment Sarcopenia With PFS

**FIGURE 4:**
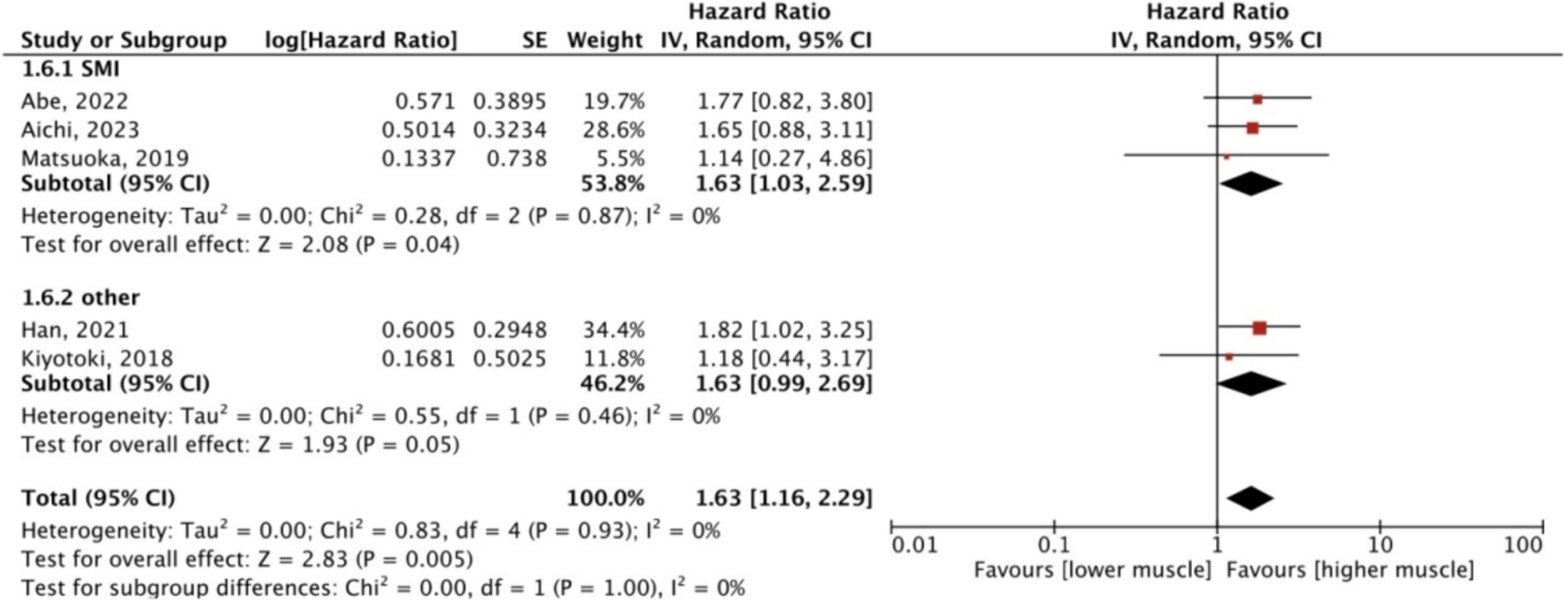
Meta‐analysis of univariate data: the effect of pretreatment sarcopenia on PFS. CI, confidence interval; df, degree of freedom; IV, inverse variance; SE, standard error.

Five studies investigated the association between sarcopenia and PFS. The meta‐analysis of univariate data on the effect of pretreatment sarcopenia on the PFS is depicted in Figure 4. The overall effect of sarcopenia on PFS was significant (HR 1.63, 95%CI: 1.16–2.29, *p* = 0.005, *I* ^*2*^ = 0). Subgroup analysis based on muscle mass assessment methods showed that sarcopenia affected PFS when SMI was adopted (*p* = 0.04), whereas the effect was insignificant when volumetric SMI or SMA was used (*p* = 0.05). Two studies by Lee [15, 21] used DRFS and DFS as assessment metrics of outcome and found different effects of low muscle mass on DRFS and DFS, with HRs of 1.823 and 1.18, 95% CIs of 1.023–3.248 and 0.44–3.17, respectively.

Han et al. [17], Aichi et al. [20] and Kise et al. [26] reported the results of multivariable regression analysis, showing a significant association between sarcopenia and PFS (HR: 1.55, 95%CI 1.06–2.28, *p* = 0.03) (Figure S2). Although Han et al. [17] used volumetric SMI as muscle mass assessment, whereas Aichi et al. [20] and Kise et al. [26] used SMI and PMI, respectively, the number of studies was too small to conduct a subgroup meta‐analysis.

### 3.4 Muscle Change and Clinical Outcomes

#### 3.4.1 Association of Muscle Loss With OS

**FIGURE 5:**
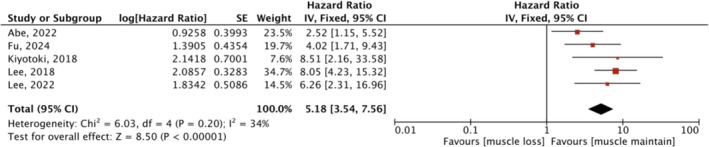
Meta‐analyses of univariate data: the effect of muscle loss during treatment on OS. CI, confidence interval; df, degree of freedom; IV, inverse variance; SE, standard error.

**FIGURE 6:**
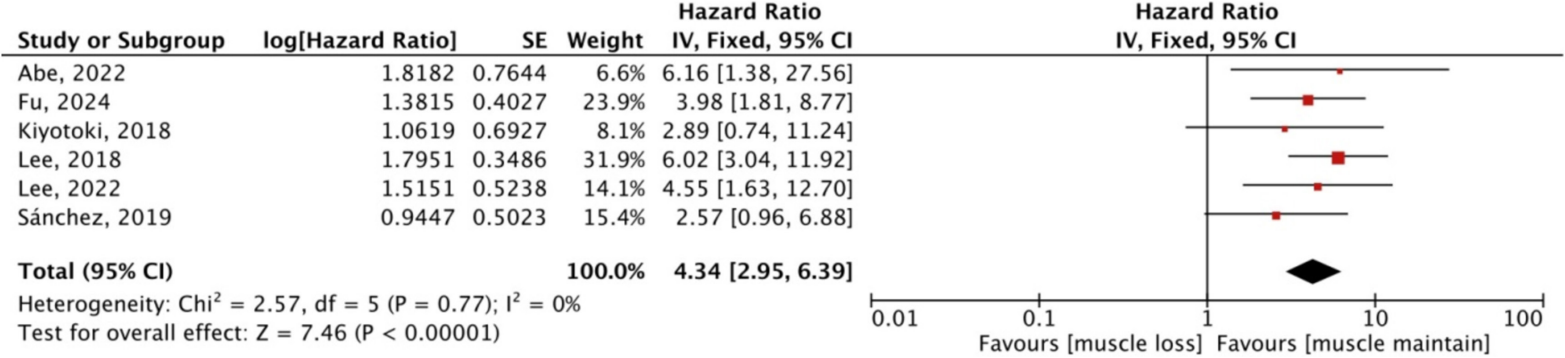
Meta‐analyses of multivariate data: the effect of muscle loss during treatment on OS. CI, confidence interval; df, degree of freedom; IV, inverse variance; SE, standard error.

Five studies investigated the association between muscle change during treatment and OS. Combining data from univariate data showed a significant association between muscle loss and poorer OS (HR 5.18, 95% CI: 3.54–7.56) (Figure 5). Among them, Kiyotoki et al. [18] and Fu et al. [28] adopted SMA and PMA changes as measurements, respectively, whereas the others used SMI changes, but the results did not change significantly after we removed these two studies (HR 5.07, 95% CI: 2.42–10.63, *p* < 0.00001). Lee et al. [15] and Abe et al. [24] used muscle mass reduction of > 5% and > 7% as cut‐offs for the presence of muscle loss, respectively, whereas the remaining three studies defined a drop in muscle mass exceeding 10% as muscle loss. The combined result did not change in different degrees of muscle loss in subgroup analysis. The meta‐analysis of multivariate data on the effect of muscle loss on OS is depicted in Figure 6, and the overall effect was significant (HR 4.34, 95% CI 2.95–6.39, *p* < 0.00001), with no heterogeneity (*I* ^*2*^ = 0). If we removed studies by Lee et al. [15] and Abe et al. [24], which set muscle loss as > 5% and > 7%, the combined result was 4.18 (95% CI: 2.71–6.45, *p* < 0.00001). Subgroup analysis according to muscle mass measurement methods confirmed consistent results (HR: 4.70, 95% CI:2.94–7.50, *p < 0.0001*, *I* ^*2*^ = 0) after removing the study by Kiyotoki et al. [18] and Fu et al. [28], which adopted SMA and PMA as indicators.

#### 3.4.2 Association of Muscle Change With PFS

**FIGURE 7:**
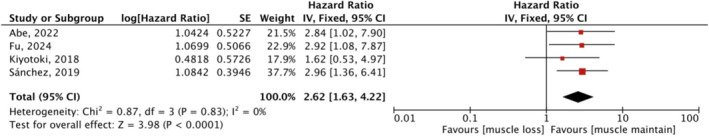
Meta‐analyses of multivariate data: the effect of muscle loss during treatment on PFS. CI, confidence interval; df, degree of freedom; IV, inverse variance; SE, standard error.

In total, the meta‐analysis of four studies showed that muscle loss during treatment was significantly associated with PFS, with the pooled HR for PFS being 2.62 (95% CI:1.63–4.22) compared with those patients with muscle maintenance (Figure 7). A fixed‐effects model was used due to *I* ^*2*^ = 0. Additionally, two studies by Lee et al. [15, 21] investigating the association between muscle loss and DFS and DRFS indicated that muscle loss was also associated with shorter DRFS and DFS.

#### 3.4.3 Sensitivity Analysis and Publication Bias

Sensitivity analysis, conducted by sequentially removing each study, confirmed the stability and reliability of the original results. Publication bias was assessed for the pretreatment sarcopenia on OS as there were fewer than 10 included studies in other comparisons [31]. The funnel plot for the effect of pretreatment sarcopenia on OS is shown in Figure S3, and Egger’s test performed by STATA showed *p* = 0.002, whereas Begg’s test showed *p* = 0.592. After Nattenmüller et al.’s study [23] was removed, the *p* values of Egger and Begg’s tests turned into 0.062 and 0.466, respectively, indicating no significant bias.

## 4 Discussion

Patients with CC are at increased risk of malnutrition due to the wasting characteristics of malignant tumours and the gastrointestinal side effects often caused by anticancer treatment. Reliable indicators are necessary to assess their nutritional status to provide appropriate nutritional support. Traditional body mass index has limitations in accurately reflecting nutritional status and body composition. Sarcopenia, a new nutritional indicator, has been recognized as a core criterion for assessing the nutritional status of patients with cancer and is closely related to clinical outcomes [4, 5]. The high prevalence of sarcopenia in patients with CC necessitates evaluating its clinical predictive value in patients. Although some studies have reported the relationship between muscle mass and clinical outcomes in patients with CC, the results are inconsistent. This systematic review and meta‐analysis is the first to summarize the existing evidence and demonstrate that pretreatment sarcopenia significantly predicted worse OS and PFS, similar to the findings in ovary cancer [32]. There was little heterogeneity in the study pooling, and the sensitivity analysis was invalidated. Subgroup analysis based on different methods of muscle mass assessment and cut‐off values influenced the association between pretreatment sarcopenia and OS and also PFS.

The method of muscle assessment is crucial for sarcopenia diagnosis and comparison between studies. The included literature uniformly used the L3 level of the CT scan to assess muscle mass, with commonly used indices including SMI (10 studies), PMI (4 studies), IM (1 study) and volumetric SMI (1 study). Subgroup analysis showed that SMI was associated with OS and PFS in both univariate and multivariate analyses, whereas other indicators may not, such as PMI. Even among studies using the same evaluation methods, the cut‐off values used to define sarcopenia varied and influenced the association between sarcopenia and clinical outcomes, but there is no official consensus. Studies with higher cut‐off values showed no significant association between sarcopenia and clinical outcomes. Notably, muscle strength and physical performance, included in the European Working Group on Sarcopenia (EWGOS 2) and Asian Working Group on Sarcopenia 2019 (AWGS 2019) guidelines [33, 34], were rarely addressed in the research. Retrospective studies assessing muscle mass via CT imaging without considering muscle strength and function ignores the multifaced complexity of sarcopenia and does not fully convey the breadth of its clinical impact among patients with cancer. Therefore, future prospective studies should comprehensively evaluate sarcopenia among patients with cancer.

The incidence of sarcopenia ranged from 26.1% to 51.8% found in this article, which might be attributed to the different cut‐off values adopted (35.6–41 cm^2^/m^2^). The SMI cut‐off values were 38.5 cm^2^/m^2^ [29] for female patients with respiratory and gastrointestinal tract cancer and 41 cm^2^/m^2^ [30] for female patients with lung cancer, but varied in gynaecological tumours. Studies with cut‐off values between 38.5 and 38.73 cm^2^/m^2^ were more likely to report a positive predictive effect of sarcopenia on clinical outcomes in ovarian cancer patients, suggesting lower cut‐off values (< 38.5 cm^2^/m^2^) for better differentiation [32]. In the studies included for pretreatment SMI predicting OS, subgroup studies with SMI thresholds of 38.5 cm^2^/m^2^ and above showed an insignificantly predictive effect of muscle mass on OS, indicating that high thresholds should be avoided, especially in Asian populations. Some studies have set cut‐off values based on tertiles, quartiles or the standard deviation of the data of the study population, which is also an acceptable method. Additionally, the selection of different muscle measurement metrics also affected the results. In Han et al.'s study [17], for instance, pretreatment SMI was not predictive of OS, but volumetric SMI was significantly associated with poor OS. In studies by Lee and others [20, 22], SMD was adopted but did not show a role in clinical outcomes in patients with CC. Studies using PMI did not find a predictive role for OS in univariate analysis, possibly due to the small area of total lumbar skeletal muscle represented by psoas.

Cancer and anticancer treatment have impacts on skeletal muscle mass, which could be a potential indicator for clinical outcomes. Observing muscle mass dynamics during anticancer treatment might be more predictive of clinical outcomes than pretreatment measurements alone. Weight changes alone may not accurately identify nutritional problems due to factors like fluid retention and increased fat mass. Studies have found an association between decreased skeletal muscle mass and survival in end‐stage pancreatic cancer [35] and Stage I–III colorectal cancer patients [36]. This meta‐analysis found that muscle mass reduction during treatment was predictive for both OS and PFS. Moreover, according to the subgroup analysis of different degrees of decrease, either a 5% or ≥ 10% decrease in muscle mass suggested adverse clinical outcomes. However, the limited number of studies investigating muscle changes in CC patients during treatment calls for more high‐quality clinical research to investigate the impact of muscle change on CC patients.

Sarcopenia is associated with poor clinical outcomes in cancer, but how it is involved in cancer prognosis remains elusive. Muscle mass is not only an indispensable structure for maintaining the activity of the organism, but it is also important for the regulation of the body's metabolism. A growing body of research confirms that low skeletal muscle mass is associated with chronic low‐grade local and systemic inflammation, independent of cancer stage, age and gender [37]. Systematic inflammation and the altered pharmacokinetics of anticancer drugs driven by low muscle mass increase the risk of anticancer treatment toxicity [38], decreasing anticancer treatment tolerance. Moreover, skeletal muscle plays a primary role in insulin‐mediated glucose metabolism. In the case of sarcopenia, the alteration of insulin sensitivity‐regulating myokine secretion [39], and lipids accumulating in muscle tissue induces insulin resistance, which was associated with overall and cancer‐specific survival. In addition, decreased muscle mass may also be related to the sensitivity of tumour cells to immunotherapy, as seen in patients with advanced non‐small cell lung cancer treated with immune checkpoint inhibitors [40].

This meta‐analysis is the first to summarize recent results to date and provide a quantitative assessment of sarcopenia and its clinical outcomes in patients with CC. Previous meta‐analyses on gynaecologic oncology included fewer studies up to 2022 and different tumour types [8, 9, 10, 11], resulting in heterogeneous findings. In comparison, this meta‐analysis includes studies up to 14 published until 2024 with 2020 participants, focusing on CC and using CT scans for muscle mass assessment, creating a relatively homogeneous cohort, enhancing the reliability of the conclusions. Additionally, changes in muscle mass may impact on clinical outcomes as patients undergo anticancer therapy. The standardized and uniform treatment methods of CC make it a good cohort to explore the effect of muscle mass changes on clinical outcomes. The present study fills this gap by including a total of 7 studies that addressed the prediction of muscle mass changes during treatment on clinical outcomes. Thus, our article provides relatively up‐to‐date and comprehensive evidence for elucidating the impact of sarcopenia on clinical outcomes in patients with CC. Moreover, our review of the works of literature also revealed the absence of muscle quality and physical performance assessment during treatment for patients with CC, which are suggested to be better predictors of adverse clinical outcomes [33]; therefore, this meta‐analysis provides suggestions for future directions of research in this field.

However, there are some limitations to this article. Firstly, except for Sánchez et al.'s studies [27], most were retrospective, and therefore, there might be some risk of bias. Secondly, the pathological types of CC include adenocarcinoma and squamous cell carcinoma, but except for Kise et al.'s study [26], none of them analysed the pathological subtypes separately; neither was the subgroup analysis presented of patients with different clinical stages, so we could not conduct subgroup analysis according to cancer stage or pathology. In the end, some studies that did not perform or report multivariate regression analyses because they did not find significant differences in clinical outcomes when grouping based on the presence of sarcopenia or had negative results on univariate regression analyses [18, 19, 21, 22, 24] thus may lead to the pooled results deviating from the actual results. Overall, CT scans have brought convenience to the objective assessment of sarcopenia in oncology patients. Prospective studies among patients with CC should be conducted to evaluate nutrition and comprehensive muscle status, including muscle strength and physical activity, to explore the impact of muscle on clinical outcomes and possible ways of nutrition intervention.

## 5 Conclusion

This systematic review and meta‐analysis indicate that pretreatment sarcopenia and muscle loss during anticancer treatment are significantly associated with poor clinical outcomes in patients with CC. Muscle mass assessment before and during treatment should be included in the routine to help clinicians and dietitians adjust therapeutic approach and provide timely nutritional support. A consensus on standardized and comprehensive muscle measurement methods, including muscle mass, strength and function, and cut‐off values according to different ethnic groups, is required to define sarcopenia and muscle loss in patients with CC.

## Conflicts of Interest

The authors declare no conflicts of interest.

## Supporting information


**Table S1** Search strategy of the systematic review

**Figure S1** Meta‐analysis of the effect of low PMI on overall survival. Abbreviations: CI, confidence interval; df, degree of freedom; IV, inverse variance; SE, standard error

**Figure S2** Meta‐analysis of multivariate data: the effect of pretreatment sarcopenia on PFS. Abbreviations: CI, confidence interval; df, degree of freedom; IV, inverse variance; SE, standard error

**Figure S3** Funnel plot of the pretreatment sarcopenia on OS
