# Supplementary material for: The Prognostic Value of Sarcopenia in Clinical Outcomes in Cervical Cancer: A Systematic Review and Meta‐Analysis
Source: J Cachexia Sarcopenia Muscle. 2025 Jan 11;16(1):e13674. doi: 10.1002/jcsm.13674 (PMC11724193; doi:10.1002/jcsm.13674)
Supplement: Supplementary file 1 — Table S1 Search strategy of the systematic review [file JCSM-16-e13674-s004.docx]

| **Supplementary Table 1. Search strategy of the systematic review** | | | | | |
| --- | --- | --- | --- | --- | --- |
| Disease key terms | | Muscle key terms | |  |  |
| 1)  2)  3)  4)  5)  6)  7)  8)  9)  10)  11)  12)  13)  14)  15)  16)  17)  18)  19)  20)  21)  22)  23)  24) | Uterine Cervical Neoplasms [MeSH]  Cervical Neoplasm Uterine. TI/AB  Neoplasm Uterine Cervical. TI/AB  Uterine Cervical Neoplasm. TI/AB  Neoplasms Cervix. TI/AB  Cervix Neoplasm. TI/AB  Neoplasm Cervix. TI/AB  Cervix Neoplasms. TI/AB  Cervical Neoplasms. TI/AB  Cervical Neoplasm. TI/AB  Neoplasms Cervical. TI/AB  Cancer of the Uterine Cervix.TI/AB.  Cancer of Cervix.TI/AB.  Cancer of the Cervix.TI/AB.  Cervix Cancer.TI/AB.  Cancer Cervix.TI/AB.  Uterine Cervical Cancer.TI/AB.  Cancer Uterine Cervical.TI/AB.  Cervical Cancer Uterine.TI/AB.  Uterine Cervical Cancers.TI/AB.  Cervical Cancer.TI/AB.  Cancer Cervical.TI/AB.  Cervical Cancers.TI/AB.  OR/ 1-23 | 25)  26)  27)  28)  29) | Sarcopenia [MeSH]  sarcopenia.TI/AB.  sarcopenias.TI/AB.  muscle.TI/AB  OR/ 25-28 | 30) | #24 AND #29 |
| **^Abbreviations/symbols: /^**^, Medical Subject Heading (MeSH) for Web of Science; TI/AB, searches fields including the Web of Science title and abstract.^  ^The present search strategy was developed for Web of Science and was modified as appropriate for other databases.^  ^Original search was conducted on July 04, 2024.^ | | | | | |
