# Supplementary material for: The Prognostic Value of Sarcopenia in Clinical Outcomes in Cervical Cancer: A Systematic Review and Meta‐Analysis
Source: J Cachexia Sarcopenia Muscle. 2025 Jan 11;16(1):e13674. doi: 10.1002/jcsm.13674 (PMC11724193; doi:10.1002/jcsm.13674)
Supplement: Supplementary file 2 — Figure S1 Meta‐analysis of the effect of low PMI on overall survival. Abbreviations: CI, confidence interval; df, degree of freedom; IV, inverse variance; SE, standard error [file JCSM-16-e13674-s001.docx]

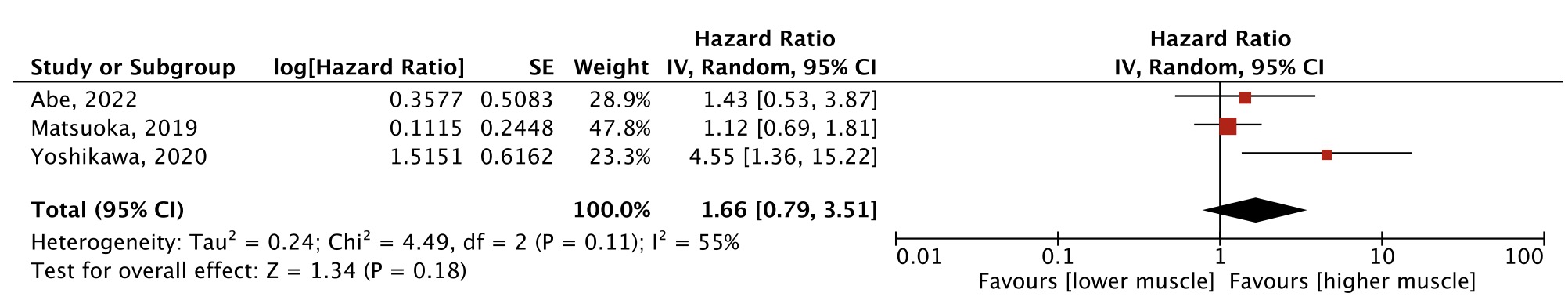


**Supplementary Fig. 1** Meta-analysis of the effect of low PMI on overall survival. Abbreviations: CI, confidence interval; df, degree of freedom; IV, inverse variance; SE, standard error
