# Supplementary figures and images for: The Prognostic Value of Sarcopenia in Clinical Outcomes in Cervical Cancer: A Systematic Review and Meta‐Analysis
Source: J Cachexia Sarcopenia Muscle. 2025 Jan 11;16(1):e13674. doi: 10.1002/jcsm.13674 (PMC11724193; doi:10.1002/jcsm.13674)

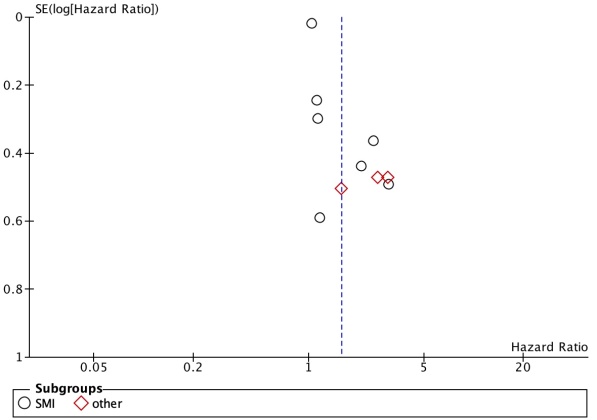


**Supplementary Fig.3** Funnel plot of the pretreatment sarcopenia on OS

Supplement: Supplementary file 4 — Figure S3 Funnel plot of the pretreatment sarcopenia on OS [file JCSM-16-e13674-s002.docx]
